# Commentary on Ivancic et al.: Enzyme kinetics from circular dichroism of insulin reveals mechanistic insights into the regulation of insulin-degrading enzyme

**DOI:** 10.1042/BSR20181555

**Published:** 2018-11-28

**Authors:** Giuseppe Grasso

**Affiliations:** Department of Chemical Sciences, University of Catania, Viale Andrea Doria 6, 95125, Catania, Italy

**Keywords:** enzyme kinetics, enzyme-substrate interactions, enzyme activity, insulin, metalloproteases, protease inhibitor

## Abstract

Despite the enormous number of therapeutic advances in medicine, nowadays many diseases are still incurable, mainly due to the lack of knowledge of the pathological biochemical pathways triggering those diseases. For this reason, it is compulsory for the scientific community to investigate and unveil the biomolecular mechanisms responsible for the development of those diseases, such as Alzheimer’s disease and diabetes, which are widespread all over the world. In this scenario, it is of paramount importance to develop new analytical techniques and experimental procedures that are capable to make the above-mentioned investigations feasible. These new methods should allow easy performable analysis carried out in a label-free environment, in order to give reliable answers to specific biochemical questions. A recent paper published on *Bioscience Reports* by Ivancic et al. (https://doi.org/10.1042/BSR20181416) proposes a new analytical technique capable to reveal some mechanistic insights into the regulation of insulin-degrading enzyme (IDE), a protein involved in the above-mentioned diseases. IDE is a multifaceted enzyme having different and not well-defined roles in the cell, but it is primarily a proteolytic enzyme capable to degrade several different amyloidogenic substrates involved in different diseases. Moreover, many molecules are responsible for IDE activity modulation so that understanding how IDE activity is regulated represents a very challenging analytical task. The new analytical approach proposed by Ivancic et al. reports on the possibility to study IDE activity in an unbiased and label-free manner, representing a valid alternative assay for the investigation of any proteases degradative activity.

Scientific progress goes hand in hand with analytical advances. Nowadays, in many fields of science, new technological and experimental approaches are necessary to achieve scientific discoveries, which, in most cases, would be impossible to attain without the right tools of investigation. This is especially valid when the investigation of complex biomolecular mechanisms has to be carried out, as setting up the appropriate assays to identify the pathological pathways responsible for the development of certain diseases is a very challenging task and false positives due to ill designed assays are always behind the corner. For example, some analytical techniques commonly used to monitor enzyme kinetics *in vivo* in order to identify regulators governing the activity of specific enzymes toward various substrates are often prone to biased or wrong results. Many enzyme activity investigations carried out with potentially biasing methods can be found in the literature, ranging from the use of fluorescent tagged substrates [1] to the presence of contaminants reported to affect the enzyme kinetics [2]. Such kinds of problems have been reported also for the investigation of insulin-degrading enzyme (IDE) activity using iodinated insulin [3].

In their work, Ivancic et al*.* [4] propose that the site-specific iodination of insulin enhances the interaction of insulin with IDE, thus making the relevance of studies using iodinated insulin to investigate IDE activity difficult to ascertain. Moreover, in IDE activity studies, things are also complicated by the fact that this enzyme is a multifaceted protein that, besides working as a degradative enzyme toward a wide range of substrates, is also capable to activate ubiquitin molecules [5] and to affect proteasome activity [6]. Therefore, the *in vivo* modulation of IDE activity has to take into account the various degradable substrates as well as the different functions of the enzyme. For example, as Ivancic et al*.* point out in their work, unspecific boosting of IDE activity in order to diminish the amyloid-β (Aβ) load and tackle Alzheimer’s disease (AD) could have deleterious consequences in the insulin metabolism; vice versa, modulating IDE activity toward insulin as a therapeutic strategy for diabetes could produce an altered homeostasis of Aβ, possibly leading to AD.

For the reasons outlined above, it is clear that scientific investigation on IDE activity could benefit from analytical methods that are capable to monitor the enzyme activity in an unbiased and substrate specific manner. The method proposed by Ivancic et al*.* is based on the conformational changes, monitored by CD, undergone by one of the IDE substrates (insulin in this case) during enzyme degradation. The method is particularly suitable in the case of insulin degradation by IDE because of the general mechanism involved with this enzyme catalytic activity: IDE is capable to degrade several different substrates, but a common feature of the latter seems to be the amyloidogenic determinant, which is the capability of the substrate to form amyloid β structures [7]. The structure of the substrate must unfold following its binding and degradation within the crypt of IDE and it becomes therefore possible to follow the enzyme kinetics simply by monitoring the CD spectrum. Such approach, besides being advantageous, as mentioned above, does not have some drawbacks of other label-free techniques such as mass spectrometry (MS), commonly used to monitor enzyme activity [8–10]. Indeed, monitoring the relative abundance of the substrate signal in a mass spectrum at different incubation time of the enzyme degradation, despite having the advantage of giving information on cleavage sites [11], has to deal with the problems related to the different ionization efficiencies of the various peptide fragments.

The absence of experimental biases of the newly proposed CD-based kinetic study allowed Ivancic et al*.* to assess IDE proteolytic activity in a substrate specific manner, focusing on insulin degradation despite the presence of other possible interfering species in solution. Indeed, the proposed assay paves the way to further enzymatic investigations where other molecules acting as IDE activity modulators are introduced. In the case of insulin and IDE, the authors found that Mg^2+^ and ATP are inefficient to change the catalytic parameters of the enzymatic reaction in contrast with what it is expected in the case of Aβ and IDE [12]. Surely, this is a clear example of the potential applicability of the newly proposed approach to study the effect of modulators, which are specific for particular enzyme–substrate systems. Such a screening represents a very useful analytical tool in the search for new drugs targeting specific enzyme activities, the new frontier of pharmaceutical research nowadays [13].

## Abbreviations

AD: Alzheimer’s disease
IDE: insulin-degrading enzyme
